# Universality and Exact Finite-Size Corrections for Spanning Trees on Cobweb and Fan Networks

**DOI:** 10.3390/e21090895

**Published:** 2019-09-15

**Authors:** Nickolay Izmailian, Ralph Kenna

**Affiliations:** 1 A. Alikhanyan National Laboratory (Yerevan Physics Institute), Alikhanian Brothers 2, Yerevan 375036, Armenia; 2 Bogoliubov Laboratory of Theoretical Physics, Joint Institute for Nuclear Research, 141980 Dubna, Russia; 3 Statistical Physics Group, Centre for Fluid and Complex Systems, Coventry University, Coventry CV1 5FB, UK; r.kenna@coventry.ac.uk

**Keywords:** universality, corrections to scaling, corners, spanning tree, cobwebm fan, Ivashkevich-Izmailian-Hu algorithm, 01.55+b, 02.10.Yn, 82B20

## Abstract

The concept of universality is a cornerstone of theories of critical phenomena. It is very well understood in most systems, especially in the thermodynamic limit. Finite-size systems present additional challenges. Even in low dimensions, universality of the edge and corner contributions to free energies and response functions is less investigated and less well understood. In particular, the question arises of how universality is maintained in correction-to-scaling in systems of the same universality class but with very different corner geometries. Two-dimensional geometries deliver the simplest such examples that can be constructed with and without corners. To investigate how the presence and absence of corners manifest universality, we analyze the spanning tree generating function on two different finite systems, namely the cobweb and fan networks. The corner free energies of these configurations have stimulated significant interest precisely because of expectations regarding their universal properties and we address how this can be delivered given that the finite-size cobweb has no corners while the fan has four. To answer, we appeal to the Ivashkevich–Izmailian–Hu approach which unifies the generating functions of distinct networks in terms of a single partition function with twisted boundary conditions. This unified approach shows that the contributions to the individual corner free energies of the fan network sum to zero so that it precisely matches that of the web. It therefore also matches conformal theory (in which the central charge is found to be c=−2) and finite-size scaling predictions. Correspondence in each case with results established by alternative means for both networks verifies the soundness of the Ivashkevich–Izmailian–Hu algorithm. Its broad range of usefulness is demonstrated by its application to hitherto unsolved problems—namely the exact asymptotic expansions of the logarithms of the generating functions and the conformal partition functions for fan and cobweb geometries. We also investigate strip geometries, again confirming the predictions of conformal field theory. Thus, the resolution of a universality puzzle demonstrates the power of the algorithm and opens up new applications in the future.

## 1. Introduction

Spanning trees are of fundamental and practical importance for connected graphs as they represent the most efficient manner in which every node in the structure can be linked. Their enumeration is a famous challenge in combinatorial graph theory originally considered by Kirchhoff in the context of electrical networks [1]. Besides being of interest in mathematics as a fundamental challenge [2,3], enumeration of such trees remains of importance to other disciplines such as physics, engineering and computing [4,5]. Diverse contexts include standard [6] and loop-erased random walks [7], and spanning tree numbers can be mapped to the partition function of the *q*-state Potts model [8] in the limit of *q* approaching zero. Another limit is v→0, where $v=e^{J}-1$, and *J* is the coupling constant, with different fixed values of v/q delivering the generating function for spanning trees, or spanning forests, etc. [9]. Another close relationship is to the sandpile model [10]. Because of this diversity of applications [11], mathematical investigations of spanning trees continue apace, building on the considerable progress already achieved. The exact numbers of spanning trees have been determined for regular lattices [4,12,13], networks [14,15] and Sierpinski gaskets [16], and the well-known bijection between close-packed dimer coverings and spanning tree configurations on two related lattices [17] add to their attractiveness as a challenging realm of study.

Systems that are confined by various boundary conditions when finite in extent have the same per-site properties in the bulk limit—e.g., free energy, internal energy, and specific heat. However, boundary characteristics are manifest in the correction terms of finite systems. The study of finite-size scaling and corrections to scaling in this context was instigated over 40 years ago ago by Fisher and Barber [18] and continues to attract a great deal of attention both for fundamental and applied purposes (see, e.g., [19,20] for well-known reviews and [21,22] for corner and boundary effects in Ising and Potts models). Theoretical interest includes extrapolating finite or partially-finite systems to determine critical and non-critical properties of their infinite counterparts. More practical interests in finite-size effects stem from recent progress in fine processing technologies for nanoscale materials with novel shapes that have recently been enabled [23,24,25]. To understand scaling and corrections terms, exact results are of prime interest because only in these cases can the analysis deliver results without the vagaries of numerical errors.

In 2002, Ivashkevich, Izmailian, and Hu [26] developed a method that delivers exact finite-size corrections for various functions of fundamental importance—e.g., partition functions and their derivatives. The method was applied to iconic models of statistical physics such as the Ising, dimer, and Gaussian models and the results demonstrated that the partition function of each model can be written as a generic partition function with twisted-boundary conditions, viz. $Z_{\alpha ,\beta }$ with (α,β)=(1/2,0),(0,1/2), and (1/2,1/2). Building upon this approach, computations by Izmailian, Oganesyan, and Hu [27] delivered finite-size corrections to the free energy of the square-lattice dimer model under five distinct sets of boundary conditions (namely free, cylindrical, and toroidal boundaries as well as the Möbius strip and the Klein bottle). The dependence of finite-size corrections on the aspect ratio was found to be sensitive to boundary conditions as well as to the parity of the number of lattice sites along the lattice axes. In 2014, Izmailian et al. [28] found that in the case of a rectangular (2M−1)×(2N−1) lattice with free and cylindrical boundary conditions, with a single monomer on the boundary, the partition functions of the anisotropic dimer model can be written in terms of a partition function with twisted-boundary conditions $Z_{\alpha ,\beta }$ with (α,β)=(0,0). Based on these considerations, the exact asymptotic expansions of the free energy were calculated.

Let us denote a connected graph by G=G(V,E), where *V* is the set of its vertices and *E* is the set of its edges. A spanning tree *T* is a subgraph of *G* which has |V|−1 edges with at least one edge at each vertex (we consider the case without loops). The number of edges attached to a vertex is its degree or coordination number. In 2000, closed-form expressions for the spanning-tree generating function were derived by Tzeng and Wu for a *d*-dimensional hypercubic lattice with free and periodic boundary conditions and for a combination of the two. Analogous results were obtained for a simple quartic net embedded on two nonorientable surfaces, namely the Klein bottle and Möbius strip [12]. In 2015, Izmailian and Kenna considered five different sets of boundary conditions for the spanning tree on finite square lattices, and expressed the partition functions in terms of a principal partition function with twisted-boundary conditions. In each case, they also derived the exact asymptotic expansions of the logarithm of the partition function [29]. Izmailian, Kenna and Wu also derived the spanning-tree-generating function for cobweb and fan networks [14,15].

In this paper, we complement the above studies by deriving expressions for the generating function of the spanning tree on cobweb and fan networks in terms of a partition function with twisted-boundary conditions $Z_{0,1/2}\left(z,M,N\right)$. The significance of this result is that it verifies the applicability of the methods and algorithms developed in Ref. [26]. To demonstrate the broad range of the usage of said the algorithm to hitherto unsolved problems, we furthermore derive the exact asymptotic expansions of the logarithm of the generating function for all networks mentioned above. In all cases we show that the exact asymptotic expansion of the free energy takes the form

(1) $$f=f_{\mathrm{bulk}}+\frac{2f_{1s}}{M}+\frac{2f_{2s}}{N}+f_{\mathrm{corn}}\frac{\ln S}{S}+\frac{f_{0}\left(\rho \right)}{S}+\sum_{p=1}^{\infty }\frac{f_{p}\left(\rho \right)}{S^{p+1}}.$$

Here, S=M×N is the area of the lattice, ρ=zξ and ξ=M/N is the aspect ratio. The term *z* is

(2) $$z=\frac{x}{y},$$

where *x* and *y* are the weights associated with the edges in the horizontal and vertical directions, respectively. The bulk free energy is $f_{\mathrm{bulk}}$, the surface free energies are $f_{1s}$ and $f_{2s}$ and the corner free energy is $f_{\mathrm{corn}}$. The leading finite-size correction term is $f_{0}\left(\rho \right)$ and the subleading correction terms are $f_{p}\left(\rho \right)$ for p=1,2,3,….

The bulk free energy term $f_{\mathrm{bulk}}$ is nonuniversal as are the surface free energies $f_{1s}$ and $f_{2s}$ and the subleading correction terms $f_{p}\left(\rho \right)$ (p=1,2,3,…). In contrast, $f_{\mathrm{corn}}$ is believed to be universal [30,31]. The leading finite-size correction term $f_{0}\left(\rho \right)$ is related to the conformal partition function and, in the limits ρ→∞ and ρ→0, its value is related to the conformal anomaly *c* and conformal weights of the underlying conformal theory [32,33]. Moreover, in 1991, Kleban and Vassileva [34] have shown that in a rectangular geometry on the plane the leading finite-size correction term $f_{0}\left(\rho \right)$ contains a geometry-dependent universal part $f_{\mathrm{univ}}\left(\rho \right)$ given by

(3) $$f_{\mathrm{univ}}\left(\rho \right)=\frac{c}{4}\ln\left[\eta (\rho )\eta \left(1/(\rho )\right)\right].$$

Here, η(ρ) is the Dedekind η function. However, Kleban and Vassileva [34] mentioned that $f_{0}\left(\rho \right)$ can also contain a non-universal additive constant $f_{\mathrm{nonuniv}}$, which is not calculable via conformal field theory methods:

(4) $$f_{0}\left(\rho \right)=f_{\mathrm{univ}}\left(\rho \right)+f_{\mathrm{nonuniv}}.$$

There is little evidence to support these predictions from either exact solutions or numerical determinations [28,29,35]. An efficient bond propagation algorithm was recently used to compute the partition function of the Ising model with free edges and corners in two dimensions on a rectangular lattice [35]. An efficient bond propagation algorithm was recently used to compute the partition function of the Ising model with free edges and corners in two dimensions on a rectangular lattice [35]. They verify the predictions of conformal field theory presented in by Equation (3) with central charge c=1/2. Later, the conformal field theory prediction Equation (3) was confirmed [28] for the dimer model on odd-odd square lattices with one monomer on the boundary, for which the central charge is c=−2 and for another model in the c=−2 universality class, i.e., the spanning-tree model with free boundary conditions [29]. Moreover, the non-universal additive constant $f_{\mathrm{nonuniv}}$ has been determined in rectangular geometry in the Ising universality class [35] and in c=−2 universality class for the dimer model [28] and spanning tree model [29]. We are not aware of any other results similar to Equation (3) for other geometries, except the plane. For example, in the torus geometry, the leading finite-size correction term $f_{0}\left(\rho \right)$ has been derived for three models on a torus in different universality classes, namely the Ising (c=1/2) [36], dimer (c=−2) [37] and Gaussian (c=1) models [26]. They take the form

(5) $$\begin{array}{ccc} f_{0}\left(\rho \right) & = & -\ln\frac{\theta_{2}+\theta_{3}+\theta_{4}}{2\eta (\rho )}\;\mathrm{for}\;\mathrm{the}\;\mathrm{Ising}\;\mathrm{model}, \end{array}$$

(6) $$\begin{array}{ccc} f_{0}\left(\rho \right) & = & -\ln\frac{\theta_{2}^{2}+\theta_{3}^{2}+\theta_{4}^{2}}{2\eta^{2}\left(\rho \right)}\;\mathrm{for}\;\mathrm{the}\;\mathrm{dimer}\;\mathrm{model}, \end{array}$$

(7) $$\begin{array}{ccc} f_{0}\left(\rho \right) & = & \ln\sqrt{\rho }\;\eta^{2}\left(\rho \right)=\ln\eta \left(\rho \right)\eta \left(1/\rho \right)\;\mathrm{for}\;\mathrm{the}\;\mathrm{Gaussian}\;\mathrm{model}. \end{array}$$

One can see from Equations (5)–(7) that in the torus geometry the leading finite-size correction term $f_{0}\left(\rho \right)$ cannot be represented in a form similar to Equation (3). Nevertheless, they are related to the conformal partition function in a torus geometry. For example, for the c=1/2 universality class, the conformal partition function on torus (*Z*) is given by (see, for example, Ref. [38], page 349)

(8) $$Z=\frac{\theta_{2}}{\eta }+\frac{\theta_{3}}{\eta }+\frac{\theta_{4}}{\eta }.$$

It is easy to see from Equations (5) and (8) that, for the Ising universality class in the torus geometry, the leading finite-size correction term $f_{0}\left(\rho \right)$ is related to the conformal partition function on the torus *Z*. The same is true for other geometries and for other universality classes.

In this paper, we derive the leading finite-size correction terms $f_{0}\left(\rho \right)$ for the spanning tree model on cobweb and fan networks using the algorithm developed in Ref. [26]. We show to which conformal partition functions they are related. We also derive the leading finite-size correction term $f_{0}\left(\rho \right)$ for the Ising model on a cobweb network to see the difference between the corresponding conformal partition functions in the c=−2 and c=1/2 universality classes. We are also especially interested in the universal corner terms $f_{\mathrm{corn}}$ because they are logarithmic. Using CFT, Cardy and Peschel predicted that a corner with an angle π/2 and two edges under free boundary conditions has

(9) $$f_{\mathrm{corn}}\left(0,0\right)=-\frac{c}{32}.$$

In this formula, *c* represents the central charge defining the universality class of the system [31]. We confirmed this in Refs. [35,39] for the square and triangular lattices with free boundary conditions. Imamura et al. [40] and Bondesan et al. [41,42] also used CFT to study the corner terms with different free boundary conditions and found that the contribution to the free energy from a corner with two edges is

(10) $$f_{\mathrm{corn}}\left(\alpha \beta \right)=\Delta_{\alpha \beta }-\frac{c}{32}.$$

This formula, where $\Delta_{\alpha \beta }$ represents the conformal weight of the boundary operator inserted at the corner, was verified in our previous work on the Ising model on the square lattice with different boundary conditions [43].

## 2. Spanning Tree on Networks

Let us consider the problem of enumerating weighted spanning trees on the M×N network. The enumeration of spanning trees involves the evaluation of the tree generating function (or partition function) $Z_{\mathrm{network}}^{\mathrm{Sp}}$

(11) $$Z_{\mathrm{network}}^{\mathrm{Sp}}\left(L;x,y\right)=\sum_{T}x^{n_{x}}y^{n_{y}},$$

where we assign weights *x* and *y*, respectively, to edges in the horizontal and vertical directions. The summation is taken over all spanning tree configurations T on L and $n_{x}$ and $n_{y}$ are the numbers of edges in the spanning tree in the respective directions.

### 2.1. Spanning Tree on the Cobweb Network

**Figure 1:**
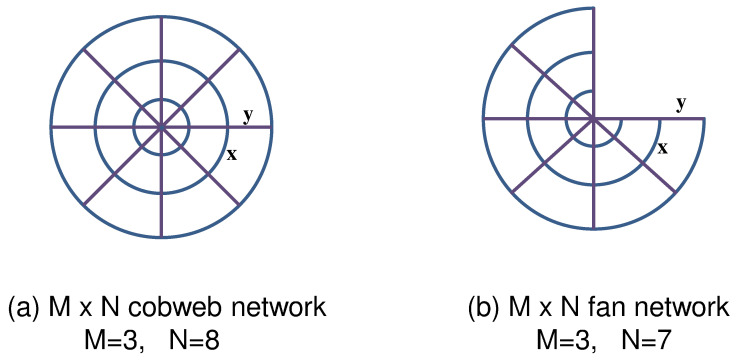
An M×N cobweb network with M=3 and N=8 (**a**). An M×N fan network with M=3 and N=7 (**b**). The weights *x* and *y* are assigned to the bonds in circular and radial directions, respectively. The cobweb network can be considered as a cylinder, where all sites on the one boundary are connected to an external common site, which is denoted by 0, while the fan network can be considered as a plane rectangular lattice, where all sites on one of four boundaries are connected to an external common site.

The cobweb lattice $L_{\mathrm{cob}}$ is an M×N rectangular lattice with periodic boundary conditions in the horizontal direction and nodes on one of the two boundaries in the other direction connected to an external common node. Therefore, there is a total of MN+1 nodes and 2MN edges. Topologically, $L_{\mathrm{cob}}$ is of the form of a wheel consisting of *N* spokes and *M* concentric circles (see Figure 1) where the circumference of the circle corresponds to the “horizontal” direction and spokes correspond to the “vertical” direction. Note that the cobweb lattice $L_{\mathrm{cob}}$ can be considered as rectangular self-dual lattices [44]. The tree generating function (or partition function) for the spanning tree model on cobweb network has been obtained in [14] and can be written as

(12) $$\begin{array}{ccc} Z_{\mathrm{cobweb}}^{\mathrm{Sp}}\left(L;x,y\right) & = & y^{MN}\prod_{n=0}^{N-1}\prod_{m=0}^{M-1}4\left[z\sin^{2}\frac{\pi n}{N}+\sin^{2}\frac{\pi (m+1/2)}{2M+1}\right], \end{array}$$

where z=x/y and *x* and *y* are weights of the edges in the spoke and circle directions, respectively.

It has been shown [26,27,29,45] that the exact partition functions of the Ising model, dimer model and spanning tree model on different planar lattices under free, cylindrical and periodic boundary conditions can be written in terms of the single expression $Z_{\alpha ,\beta }\left(z,M,N\right)$ with (α,β)=(1/2,0),(0,1/2) and (1/2,1/2), where

(13) $$Z_{\alpha ,\beta }^{2}\left(z,M,N\right)=\prod_{m=0}^{M-1}\prod_{n=0}^{N-1}4\left[z\sin^{2}\frac{(n+\alpha )\pi }{N}+\sin^{2}\frac{(m+\beta )\pi }{M}\right],$$

with (α,β)≠(0,0). Note that the general theory about the asymptotic expansion of $Z_{\alpha ,\beta }\left(z,M,N\right)$ has been given in [26,27].

In what follows, we will show that the tree generating function on the cobweb network can be expressed in terms of $Z_{0,1/2}\left(z,M,N\right)$ with M=2M+1 and N=N

(14) $$Z_{0,1/2}^{2}\left(z,2M+1,N\right)=\prod_{n=0}^{N-1}\prod_{m=0}^{2M}4\left[z\;\sin^{2}\frac{n\pi }{N}+\sin^{2}\frac{(m+1/2)\pi }{2M+1}\right].$$

First, we express double products $\prod_{n=0}^{N-1}\prod_{m=0}^{2M}f\left(n,m\right)$ in terms of $\prod_{n=0}^{N-1}\prod_{m=0}^{M-1}f\left(n,m\right)$, where

(15) $$f\left(n,m\right)=4\left[z\;\sin^{2}\frac{n\pi }{N}+\sin^{2}\frac{(m+1/2)\pi }{2M+1}\right].$$

It is easy to show that

(16) $$\prod_{n=0}^{N-1}\prod_{m=0}^{2M}f\left(n,m\right)=\left(\prod_{n=0}^{N-1}f\left(n,M\right)\right){\left(\prod_{n=0}^{N-1}\prod_{m=0}^{M-1}f\left(n,m\right)\right)}^{2},$$

with $f\left(n,M\right)=4\left(z\;\sin^{2}\frac{n\pi }{N}+1\right)$.

With the help of the identity [46]

(17) $$\begin{array}{ccc} \prod_{k=0}^{K-1}4\left[\;\;\sinh^{2}\omega +\sin^{2}\frac{k\pi }{K}\right] & = & 4\sinh^{2}\left(K\;\omega \right), \end{array}$$

the product $\prod_{n=0}^{N-1}f\left(n,M\right)$ can be written as

(18) $$\begin{array}{ccc} \prod_{n=0}^{N-1}f\left(n,M\right) & = & 4z^{N}\sinh^{2}\left[N\mathrm{arcsinh}\;\sqrt{1/z}\right]. \end{array}$$

Now, using Equations (12)–(16) and (18), the tree generating function on the cobweb network can be expressed finally as

(19) $$\begin{array}{c} Z_{\mathrm{cobweb}}^{\mathrm{Sp}}\left(L;x,y\right)=Q_{1}\;Z_{0,1/2}\left(z,2M+1,N\right), \end{array}$$

with

(20) $$\begin{array}{c} Q_{1}=\frac{y^{MN}}{2z^{N/2}\sinh\left[N\mathrm{arcsinh}\;\sqrt{1/z}\right]}. \end{array}$$

Thus, we have linked the cobweb partition function to the more general expression $Z_{\alpha ,\beta }\left(z,M,N\right)$, further extending the applicability of the latter.

### 2.2. Spanning Tree on the Fan Network

The fan lattice $L_{\mathrm{fan}}$ is an M×N rectangular lattice of *M* rows and *N* columns with free boundary conditions on three sides of the lattice and nodes on the fourth boundary connected to an external additional node. Therefore, there is a total of MN+1 nodes and 2MN−M edges. We use the term Dirichlet–Neumann to describe the boundary conditions along the fourth boundary. Topologically, the obtained lattice is of the form of “fan” consisting of *N* radial lines and *M* transverse arcs (see Figure 1), where transverse arcs correspond to the “horizontal” direction and radial lines correspond to the “vertical” directions. In other words, we impose Neumann or free boundary conditions along the two border spokes and along the outermost arc. We use the term Dirichlet–Neumann to describe the boundary conditions along the innermost arc.

The tree generating function for the spanning tree model on the fan network has been obtained in [14] and can be written as

(21) $$\begin{array}{ccc} Z_{\mathrm{fan}}^{\mathrm{Sp}}\left(L;x,y\right) & = & y^{MN}\prod_{m=0}^{M-1}\prod_{n=0}^{N-1}4\left[z\;\sin^{2}\frac{\pi n}{2N}+\sin^{2}\frac{\pi (m+1/2)}{2M+1}\right], \end{array}$$

where z=x/y.

In what follows, we will show that the tree generating function on the fan network can be expressed in terms of the single quantity $Z_{0,1/2}\left(z,2M+1,2N\right)$

(22) $$Z_{0,1/2}^{2}\left(z,2M+1,2N\right)=\prod_{n=0}^{2N-1}\prod_{m=0}^{2M}4\left[z\;\sin^{2}\frac{n\pi }{2N}+\sin^{2}\frac{(m+1/2)\pi }{2M+1}\right].$$

Now, we first express double products $\prod_{n=0}^{2N-1}\prod_{m=0}^{2M}f\left(n,m\right)$ in terms of $\prod_{n=0}^{N-1}\prod_{m=0}^{M-1}f\left(n,m\right)$, where

(23) $$f\left(n,m\right)=4\left[z\;\sin^{2}\frac{n\pi }{2N}+\sin^{2}\frac{(m+1/2)\pi }{2M+1}\right].$$

It is easy to show that f(2N−n,m)=f(n,2M−m)=f(n,m) and thus

(24) $$\prod_{n=0}^{2N-1}\prod_{m=0}^{2M}f\left(n,m\right)=\frac{\prod_{n=0}^{N-1}f{\left(n,M\right)}^{2}\prod_{m=0}^{2M}f\left(N,m\right)}{\prod_{m=0}^{2M}f\left(0,m\right)}{\left(\prod_{n=0}^{N-1}\prod_{m=0}^{M-1}f\left(n,m\right)\right)}^{4}.$$

With the help of the identities [46]

(25) $$\begin{array}{ccc} \prod_{k=0}^{K-1}4\left[\;\;\sinh^{2}\omega +\sin^{2}\frac{k\pi }{K}\right] & = & 4\sinh^{2}\left(K\;\omega \right), \end{array}$$

(26) $$\begin{array}{ccc} \prod_{k=0}^{K-1}4\left[\;\;\sinh^{2}\omega +\sin^{2}\frac{(k+1/2)\pi }{K}\right] & = & 4\cosh^{2}\left(K\;\omega \right), \end{array}$$

(27) $$\begin{array}{ccc} \prod_{m=0}^{M-1}4\sin^{2}\frac{(m+1/2)\pi }{M} & = & 4, \end{array}$$

the products $\prod_{m=0}^{2M}f\left(N,m\right)$, $\prod_{n=0}^{N-1}f{\left(n,M\right)}^{2}$ and $\prod_{m=0}^{2M}f\left(0,m\right)$ can be written as

(28) $$\begin{array}{ccc} \prod_{n=0}^{N-1}f{\left(n,M\right)}^{2} & = & \frac{4z^{2N}}{1+z}\sinh^{2}\left[2N\mathrm{arcsinh}\;\sqrt{1/z}\right] \end{array}$$

(29) $$\begin{array}{ccc} \prod_{m=0}^{2M}f\left(N,m\right) & = & 4\cosh^{2}\left[\left(2M+1\right)\mathrm{arcsinh}\;\sqrt{z}\right], \end{array}$$

(30) $$\begin{array}{ccc} \prod_{m=0}^{2M}f\left(0,m\right) & = & 4. \end{array}$$

Now using Equations (21)–(24), (28)–(30) the tree generating function on the fan network can finally be expressed as

(31) $$\begin{array}{c} Z_{\mathrm{fan}}^{\mathrm{Sp}}\left(L;x,y\right)=Q_{2}\;Z_{0,1/2}^{1/2}\left(z,2M+1,2N\right), \end{array}$$

with

(32) $$\begin{array}{c} Q_{2}=\frac{y^{MN}{\left(1+z\right)}^{1/4}}{\sqrt{2z^{N}\sinh\left[2N\mathrm{arcsinh}\;\sqrt{1/z}\right]\cosh\left[\left(2M+1\right)\mathrm{arcsinh}\;\sqrt{z}\right]}}. \end{array}$$

Again, we have extended the applicability of the general expression $Z_{\alpha ,\beta }\left(z,M,N\right)$ by linking it to the fan partition function.

## 3. Asymptotic Expansion of Free Energy

Thus, we have expressed the generating functions of the spanning tree on cobweb and fan networks in terms of a principal partition function with twisted-boundary conditions $Z_{0,1/2}\left(z,M,N\right)$ only. Based on such results, one can use the exact asymptotic expansions of $Z_{0,1/2}\left(z,M,N\right)$ given in Ref. [26] to derive the exact asymptotic expansions of the free energy of the spanning tree $f=-\frac{1}{S}\ln Z$ for all networks mentioned above in terms of the Kronecker’s double series [26], which are directly related to elliptic θ functions. For the reader’s convenience, the asymptotic expansion of $\ln Z_{0,1/2}\left(z,M,N\right)$ is given in Appendix A.

Using Equation (A3), we can easily write down all the terms of the exact asymptotic expansion Equation (1) of the free energy, $f=-\frac{1}{S}\ln Z$ for all models under consideration.

The bulk free energy $f_{\mathrm{bulk}}$ in Equation (1) for the weighted spanning tree on finite M×N+1 lattices for all networks is given by

(33) $$\begin{array}{ccc} f_{\mathrm{bulk}} & = & -\ln y-\frac{2}{\pi }\int_{0}^{\pi }\omega_{z}\left(k\right)dk=-\ln y-\frac{1}{\pi }\sum_{n=0}^{\infty }{\left(-1\right)}^{n}{\left(n+1/2\right)}^{-2}z^{n+1/2}\\ \; & = & -\ln y-\frac{z^{1/2}\;\Phi \left(-z,2,\frac{1}{2}\right)}{\pi }, \end{array}$$

where $\omega_{z}\left(k\right)$ is given by Equation (A2) and Φ(−z,2,1/2) is the Lerch transcendent. In particular, for isotropic spanning tree (z=1), the Lerch transcendent is now Φ(−1,2,1/2)=4G, where G=0.915965594… is the Catalan constant.

### 3.1. Asymptotic Expansion of Free Energy of the Spanning Tree on the Cobweb Network

Using Equations (19), (20) and (A3), the exact asymptotic expansions of the free energy for the spanning tree on the cobweb network, $f=-\frac{1}{S}\ln Z_{\mathrm{cobweb}}^{\mathrm{Sp}}$ can be written as

(34) $$\begin{array}{ccc} f & = & -\frac{1}{S}\ln Z_{\mathrm{cobweb}}^{\mathrm{Sp}}=-\ln y+\frac{\frac{1}{2}\ln x+\mathrm{arcsinh}\;\frac{1}{\sqrt{z}}}{\left(M+\frac{1}{2}\right)}-\frac{1}{S}\ln Z_{0,1/2}\left(z,2M+1,N\right)\\ \; & = & f_{\mathrm{bulk}}+\frac{2f_{1s}}{\left(M+\frac{1}{2}\right)}-\frac{1}{S}\ln\frac{\theta_{2}\left(2\;\sqrt{z}\;\xi \right)}{\eta (2\;\sqrt{z}\;\xi )}+\frac{4\pi \xi }{S}\sum_{p=1}^{\infty }{\left(\frac{\pi^{2}\xi }{S}\right)}^{p}\frac{\Lambda_{2p}}{(2p)!}\frac{K_{2p+2}^{\frac{1}{2},0}\left(2\;i\;\sqrt{z}\;\xi \right)}{2p+2}, \end{array}$$

where $f_{\mathrm{bulk}}$ is given by Equation (33), the surface free energy $f_{2s}$ is equal to zero and $f_{1s}$ is given by

(35) $$f_{1s}=\frac{1}{4}\ln x+\frac{1}{2}\mathrm{arcsinh}\;\frac{1}{\sqrt{z}},$$

and *S* and ξ are given by

(36) $$S=\left(M+\frac{1}{2}\right)N,\;\xi =\frac{M+\frac{1}{2}}{N}.$$

The expression for *S* is neither equal to the number of nodes or of edges; instead, it emerges from the asymptotic expansion of the logarithm of $Z_{0,1/2}\left(z,2M+1,N\right)$. Thus, the exact asymptotic expansions of the free energy for the spanning tree on the cobweb network can be written in the form given by Equation (1). For the leading correction terms $f_{0}\left(z\xi \right),$ we obtain

(37) $$\begin{array}{ccc} f_{0}\left(z\xi \right) & = & -\ln\frac{\theta_{2}\left(2\sqrt{z}\;\xi \right)}{\eta (2\sqrt{z}\;\xi )}. \end{array}$$

To check whether or not the leading finite-size correction term $f_{0}\left(\rho \right)$ given by Equation (37) can be represented in a form similar to Equation (3), we have to consider the Ising model (c=1/2) on the cobweb network [8]. We have obtained that the exact asymptotic expansions of the free energy for the Ising model on the cobweb network can be written in the form given by Equation (1) with z=1 (the details of the calculation will be reported elsewhere). The bulk free energy $f_{\mathrm{bulk}}$ for the Ising model on the cobweb network is given by

(38) $$f_{\mathrm{bulk}}=-\frac{1}{2}\ln2-\frac{2G}{\pi }.$$

The surface free energy $f_{2s}$ is equal to zero and $f_{1s}$ is given by

(39) $$f_{1s}=\frac{1}{2}\ln\left(1+\sqrt{2}\right)-\frac{1}{8}\ln2-\frac{1}{4\pi }\int_{0}^{\pi }\ln\left(\sqrt{2}\sin x+\sqrt{1+\sin^{2}x}\right)dx,$$

and the leading finite-size correction term $f_{0}\left(\xi \right)$ for the Ising model in the rectangular geometry on the cobweb network is given by

(40) $$f_{0}\left(\xi \right)=-\frac{1}{2}\ln\frac{2\;\theta_{4}\left(2\;\xi \right)}{\eta (2\;\xi )}.$$

Notet hat *S* and ξ for Ising model on the cobweb network are also given by Equation (36).

Thus, we can see from Equations (37) and (40) that the leading finite-size correction term $f_{0}\left(\xi \right)$ cannot be represented in a form similar to Equation (3). Instead, based on Equations (37) and (40), we can predict that the conformal partition function on the cobweb network for the Ising (c=1/2) universality class $Z_{\mathrm{Ising}}$ is given by

(41) $$Z_{\mathrm{Ising}}=\sqrt{\frac{2\;\theta_{4}\left(2\;\xi \right)}{\eta (2\;\xi )}}$$

and, for the spanning tree (c=−2) universality class, the conformal partition function on cobweb network $Z_{\mathrm{Sp}}^{\mathrm{cobweb}}$ is given by

(42) $$Z_{\mathrm{Sp}}^{\mathrm{cobweb}}=\frac{\theta_{2}\left(2\sqrt{z}\;\xi \right)}{\eta (2\sqrt{z}\;\xi )}.$$

For subleading correction terms $f_{p}\left(z\xi \right)$ for p=1,2,3,…, we get

$$\begin{array}{ccc} f_{p}\left(z\;\xi \right) & = & \frac{2\pi^{2p+1}\xi^{p+1}}{\left(2p)!(p+1\right)}\Lambda_{2p}\;K_{2p+2}^{0,1/2}\left(2i\sqrt{z}\;\xi \right). \end{array}$$

The coefficients $\Lambda_{2p}$ are given in Equation (A7) and the Kronecker’s double series $K_{2p+2}^{0,1/2}\left(iz\xi \right)$ in terms of the elliptic theta functions are given in [26,27] for arbitrary *p*.

It is easy to see from Equation (34) that the exact asymptotic expansions of the free energy for the spanning tree on the finite cobweb network do not contain the corner free energy $f_{\mathrm{corner}}$, as it should be, since the logarithmic corner corrections to the free energy density should be absent for the systems without corners.

### 3.2. Asymptotic Expansion of Free Energy of the Spanning Tree on the Fan Network

Using Equations (31), (32) and (A3), the exact asymptotic expansions of the free energy for the spanning tree on the fan network, $f=-\frac{1}{S}\ln Z_{\mathrm{fan}}^{\mathrm{Sp}}$ can be written as

(43) $$\begin{array}{ccc} f & = & -\frac{1}{S}\ln Z_{\mathrm{fan}}^{\mathrm{Sp}}\\ \; & = & -\ln y+\frac{\frac{1}{2}\ln x+\mathrm{arcsinh}\;\frac{1}{\sqrt{z}}}{\left(M+\frac{1}{2}\right)}+\frac{\mathrm{arcsinh}\;\sqrt{z}}{N}-\frac{1}{4S}\ln4\left(1+z\right)-\frac{1}{2S}\ln Z_{0,1/2}\left(z,2M+1,2N\right)\\ \; & = & f_{\mathrm{bulk}}+\frac{2f_{1s}}{\left(M+\frac{1}{2}\right)}+\frac{2f_{2s}}{N}-\frac{1}{4S}\ln4(1+z)-\frac{1}{2S}\ln\frac{\theta_{2}\left(\sqrt{z}\;\xi \right)}{\eta (\sqrt{z}\;\xi )}\\ \; & + & \frac{\pi \xi }{S}\sum_{p=1}^{\infty }{\left(\frac{\pi^{2}\xi }{4S}\right)}^{p}\frac{\Lambda_{2p}}{(2p)!}\frac{K_{2p+2}^{0,\frac{1}{2}}\left(i\sqrt{z}\;\xi \right)}{2p+2}, \end{array}$$

where $f_{\mathrm{bulk}}$ is given by Equation (33), the surface free energies $f_{1s}$ is given by Equation (35) and $f_{2s}$ is given by

(44) $$f_{2s}=\frac{1}{2}\mathrm{arcsinh}\;\sqrt{z},$$

and *S* and ξ are again given by Equation (36). The exact asymptotic expansions of the free energy for the spanning tree on the fan network can again be written in the form given by Equation (1). For the leading correction terms $f_{0}\left(z\xi \right),$ we obtain

(45) $$\begin{array}{ccc} f_{0}\left(z\xi \right) & = & -\frac{1}{4}\ln4\left(1+z\right)-\frac{1}{2}\ln\frac{\theta_{2}\left(\sqrt{z}\;\xi \right)}{\eta (\sqrt{z}\;\xi )}. \end{array}$$

Thus, from Equation (45), one can see that a geometry-dependent universal part of the free energy $f_{\mathrm{univ}}\left(z\xi \right)$ in the rectangular geometry on the fan network is given by

(46) $$f_{\mathrm{univ}}\left(z\xi \right)=-\frac{1}{2}\ln\frac{\theta_{2}\left(\sqrt{z}\;\xi \right)}{\eta (\sqrt{z}\;\xi )},$$

while a non-universal additive constant $f_{\mathrm{nonuniv}}$ is given by

(47) $$f_{\mathrm{nonuniv}}=-\frac{1}{4}\ln4\left(1+z\right).$$

As for the case of the cobweb network, we can predict that the conformal partition function for c=−2 universality class on the fan network $Z_{\mathrm{Sp}}^{\mathrm{fan}}$ is given by

(48) $$Z_{Sp}^{\mathrm{fan}}=\sqrt{\frac{\theta_{2}\left(\sqrt{z}\;\xi \right)}{\eta (\sqrt{z}\;\xi )}}.$$

It will be interesting to check whether or not the leading finite-size correction term $f_{0}\left(\sqrt{z}\;\xi \right)$ given by Equation (45) can be represented in a form similar to Equation (3) by considering models in different universality classes on the fan network, as well as to compute that term by the conformal field theory method.

For subleading correction terms $f_{p}\left(z\xi \right)$ for p=1,2,3,…, we get

(49) $$\begin{array}{ccc} f_{p}\left(z\;\xi \right) & = & \frac{\pi^{2p+1}\xi^{p+1}}{4^{p}\left(2p\right)!\left(2p+2\right)}\Lambda_{2p}\;K_{2p+2}^{0,1/2}\left(i\sqrt{z}\;\xi \right). \end{array}$$

The coefficients $\Lambda_{2p}$ are given in Equation (A7) and Kronecker’s double series $K_{2p+2}^{0,1/2}\left(iz\xi \right)$ in terms of the elliptic theta functions are given in [26,27].

Since the fan network is the plane rectangular lattice with three free boundary conditions and one with Dirichlet–Neumann boundary conditions, we have four corners for the fan network and one can expect the corner free energy $f_{\mathrm{corner}}$ contribution in the exact asymptotic expansions of the free energy Equation (43). However, it is easy to see from Equation (43) that, in the exact asymptotic expansions of the free energy for the spanning tree on the finite fan network, the corner free energy $f_{\mathrm{corner}}$ is equal to zero. Let us consider the corners of the fan network. Two of these corners have two edges each of which are subject to free boundary conditions and two corners have two edges of which one is under free and the other under Dirichlet–Neumann boundary conditions. Thus, the corner free energy $f_{\mathrm{corner}}$ can be written as a sum of four corner contribution, namely

(50) $$f_{\mathrm{corner}}=2f_{\mathrm{corn}}\left(0,0\right)+2f_{\mathrm{corn}}\left(0,\beta \right),$$

where $f_{\mathrm{corn}}\left(0,0\right)$ is the contribution to the free energy from the corner with two edges with free boundary conditions, which is given by Equation (9) with central charge *c* equal to c=−2, namely

$$f_{\mathrm{corn}}\left(0,0\right)=\frac{1}{16}$$

and $f_{\mathrm{corn}}\left(0,\beta \right)$ is the contribution to the free energy from the corner with two edges one under free and another under Dirichlet–Neumann boundary conditions, which is given by Equation (10) with central charge c=−2 and $\Delta_{0,\beta }=-1/8$. Here, $\Delta_{0,\beta }=-1/8$ is the conformal weight of the boundary operator inserted at that corner. Thus, $f_{\mathrm{corn}}\left(0,\beta \right)$ is equal to

$$f_{\mathrm{corn}}\left(0,\beta \right)=\Delta_{0,\beta }-\frac{c}{32}=-\frac{1}{16}$$

and the total contribution from the corners to free energy $f_{\mathrm{corner}}$ is equal to zero. Although the fan network has four corners and each of these gives corner contributions to the free energy, the sum of these contributions ($f_{\mathrm{corner}}$) is equal to zero. Thus, our results confirm both conformal theory [31,40,41,42] and finite-size scaling [47] predictions.

## 4. Spanning Tree on Infinitely Long Strips

Finally, let us consider the case of an infinitely long strip. Conformal invariance implies that, for an infinitely long two-dimensional (2D) strip of finite width *L* at criticality, the finite size scaling behavior of the critical free energy *f* has the form [32,33]

(51) $$f=f_{\mathrm{bulk}}+\frac{2f_{\mathrm{surf}}}{L}+\frac{A}{L^{2}}+O\left(L^{-3}\right),$$

where the bulk free energy density $f_{\mathrm{bulk}}$ and the surface free energy $f_{\mathrm{surf}}$ are nonuniversal constants. In contrast, *A* is a universal constant, but may depend on the boundary conditions. In some 2D geometries, the values of *A* are known [32,33,48] to be related to the central charge (c) and the conformal weight of the ground state Δ

(52) $$\begin{array}{ccc} A & = & 4\pi \zeta \left(\Delta -\frac{c}{24}\right)\;\;\mathrm{in}\;\mathrm{cylinder}\;\mathrm{geometry}, \end{array}$$

(53) $$\begin{array}{ccc} A & = & \pi \zeta \left(\Delta -\frac{c}{24}\right)\;\;\mathrm{in}\;\mathrm{strip}\;\mathrm{geometry}, \end{array}$$

where the anisotropy factor ζ is a nonuniversal constant.

Let us consider the spanning tree case on the cobweb and fan network in the case when M→∞ (or ξ→∞). In that case, the cobweb becomes an infinitely long cylinder with circumference *N* and the fan network becomes an infinitely long strip of width *N* and with free boundary condition on both sides of the strip. The asymptotic expansion of the free energy for the cobweb and fan networks can be obtained from Equations (34) and (43), respectively. Using the facts that

(54) $$\begin{array}{ccc} \lim_{\xi \to \infty }\theta_{2}\left(\sqrt{z}\;\xi \right) & = & \lim_{\xi \to \infty }2e^{-\frac{\pi \sqrt{z}\xi }{4}}=0, \end{array}$$

(55) $$\begin{array}{ccc} \lim_{\xi \to \infty }\theta_{4}\left(\sqrt{z}\;\xi \right) & = & \lim_{\xi \to \infty }\theta_{3}\left(\sqrt{z}\;\xi \right)=1, \end{array}$$

(56) $$\begin{array}{ccc} \lim_{\xi \to \infty }\eta \left(\sqrt{z}\;\xi \right) & = & \lim_{\xi \to \infty }e^{-\frac{\pi \sqrt{z}\xi }{12}}=0, \end{array}$$

the asymptotic expansion of the free energy for the cobweb network on the infinitely long cylinder with circumference *N* can be obtained from Equation (34)

(57) $$f=f_{\mathrm{bulk}}+\frac{\pi \sqrt{z}}{3N^{2}}+\ldots .$$

Thus, by choosing L=N, the anisotropy factor $\zeta =\sqrt{z}$, the central charge c=−2 and conformal weight of the ground state Δ=0, we get full agreement with conformal field predictions for cylinder geometry given by Equation (50).

Again, using Equations (53) and (55), the asymptotic expansion of the free energy for the fan network on the infinitely long strip with width *N* and with free boundary condition on both sides of the strip can be obtained from Equation (43)

(58) $$f=f_{\mathrm{bulk}}+\frac{2f_{2s}}{N}+\frac{\pi \sqrt{z}}{12N^{2}}+\ldots ,$$

where $f_{2s}$ is given by Equation (43). Thus, by choosing L=N, the anisotropy factor $\zeta =\sqrt{z}$, the central charge c=−2 and conformal weight of the ground state Δ=0, we get full agreement with conformal field predictions for strip geometry given by Equation (52).

Let us now consider the spanning tree case on the cobweb and fan networks in the case when N→∞ (or ξ→0). In that case, both the cobweb and fan network become infinitely long strips with width *M* and with free boundary condition on one side of the strip and Dirichlet–Neumann boundary conditions on another side of the strip. The asymptotic expansion of the free energy for the cobweb and fan networks can be obtained from Equations (34) and (43), respectively.

To obtain the asymptotic expansion of the free energy for cobweb and fan networks, we need the behavior of the $\theta_{2}\left(\tau \right)$-function and Dedekind’s η(τ)-function under the Jacobi transformation

$$\tau \to \tau^{\prime }=-1/\tau .$$

The result for the $\theta_{2}\left(\tau \right)$-functions and Dedekind’s η(τ) function is given in Appendix A of Ref. [49]:

(59) $$\begin{array}{ccc} \theta_{2}\left(\tau^{\prime }\right) & = & {\left(-i\tau \right)}^{1/2}\theta_{4}\left(\tau \right), \end{array}$$

(60) $$\begin{array}{ccc} \eta (\tau^{\prime }) & = & {\left(-i\tau \right)}^{1/2}\eta \left(\tau \right). \end{array}$$

Using Equations (54), (55), (58) and (59), one can obtain the asymptotic behavior of $\theta_{2}\left(\tau^{\prime }\right)$ and $\eta (\tau^{\prime })$ as $\tau^{\prime }\to 0$ (or N→∞). Then, from Equations (34) and (43), one can obtain the asymptotic expansion of the free energy for the cobweb and fan networks on the infinitely long strip in the following form

(61) $$f=f_{\mathrm{bulk}}+\frac{2f_{1s}}{M+1/2}-\frac{\pi }{24\sqrt{z}{\left(M+1/2\right)}^{2}}+\ldots ,$$

where $f_{1s}$ is given by Equation (35). Thus, by choosing L=M+1/2, the anisotropy factor $\zeta =1/\sqrt{z}$, the central charge c=−2 and conformal weight of the ground state Δ=−1/8, we will get full agreement with conformal field predictions given by Equations (50) and (52).

## 5. Conclusions

We analyzed spanning-tree generating functions for finite-size cobweb and fan networks and showed that each can be expressed in terms of a single, unifying partition function with twisted boundary conditions. This reveals that the four corner free energies of the fan network cancel each other out so that their sum matches the vanishing total value for the cobweb (which has no corners).

Thus, we have extended the applicability of the twisted-boundary-method to a broad set of circumstances, opening up possible new approaches to efficiently investigate a multitude of spanning-tree problems of both fundamental and practical relevance. For example, one could work backwards and seek different models which fit to the same $Z_{\alpha ,\beta }$ and thus obey a class of “strong” universality. To further demonstrate the power of the approach, we have used it to derive exact finite-size corrections for the logarithm of the generating function of the spanning tree on both of these networks. Then, based on the unified partition functions, we derived the exact asymptotic expansion of the logarithm of the partition function for the spanning tree on the cobweb and fan networks. We also explain in the context of conformal field theory why the corner free energy for fan network, with its four corners, is equal to zero. Based on our results for the leading finite-size correction term $f_{0}$ for the fan and cobweb networks, we have predicted the conformal partition functions in a c=−2 universality class for fan and cobweb geometries. For the Ising model, we have also predicted the conformal partition functions in the Ising (c=1/2) universality class for cobweb geometries. In addition, finally, we have investigated the strip geometry for both of the above-mentioned models and find an excellent agreement with conformal field theory predictions. Thus, we have confirmed universality for spanning trees and affirmed the robustness of the twisted-boundary-condition approach, opening new possible conduits to future research in a long-standing field of interest for a number of disciplines.

## Appendix A. Asymptotic Expansion of and $Z_{0,\frac{1}{2}}\left(z,M,N\right)$

For the convenience of the reader, in this appendix, we present the exact asymptotic expansions of the logarithm of $Z_{0,\frac{1}{2}}\left(z,M,N\right)$ given in Ref. [26].

With the help of the identity,

$$4{\left|\;\;\sinh\left(M\omega +i\pi \beta \right)\right|}^{2}=4\left[\;\sinh^{2}M\omega +\sin^{2}\pi \beta \;\right]=\prod_{m=0}^{M-1}4\left[\;\;\sinh^{2}\omega +\sin^{2}\left(\frac{\pi (m+\beta )}{M}\right)\right],$$

the partition function with twisted boundary conditions $Z_{\alpha ,\beta }\left(z,M,N\right)$ given by Equation (13) can be transformed into the simpler form

(A1) $$Z_{\alpha ,\beta }\left(z,M,N\right)=\prod_{n=0}^{N-1}2\left|\;\;\sinh\left[M\omega_{z}\;\left(\frac{\pi (n+\alpha )}{N}\right)+i\pi \beta \right]\right|,$$

where lattice dispersion relation has been used

(A2) $$\omega_{z}\left(x\right)=\mathrm{arcsinh}\left(\sqrt{z}\sin x\right).$$

The exact asymptotic expansion of the logarithm of $Z_{0,\frac{1}{2}}\left(z,M,N\right)$ in terms of the Kronecker’s double series [26,50] can be written as

(A3) $$\begin{array}{ccc} \ln Z_{0,\frac{1}{2}}\left(z,M,N\right) & = & \frac{S}{\pi }\int_{0}^{\pi }\omega_{z}\left(x\right)dx+\ln\frac{\theta_{2}\left(\sqrt{z}\rho \right)}{\eta (\sqrt{z}\rho )}-2\pi \rho \sum_{p=1}^{\infty }{\left(\frac{\pi^{2}\rho }{S}\right)}^{p}\frac{\Lambda_{2p}}{(2p)!}\frac{K_{2p+2}^{0,\frac{1}{2}}\left(i\sqrt{z}\rho \right)}{2p+2}, \end{array}$$

where S=MN, ρ=M/N, η(τ) is the Dedekind - η function

(A4) $$\eta \left(\tau \right)=e^{\pi i\tau /12}\prod_{n=1}^{\infty }\left(1-e^{2\pi i\tau n}\right).$$

Dedekind’s η function satisfies the following identity:

(A5) $$2\eta {\left(\tau \right)}^{3}=\theta_{2}\left(\tau \right)\theta_{3}\left(\tau \right)\theta_{4}\left(\tau \right),$$

where $\theta_{2}\left(\tau \right),\theta_{3}\left(\tau \right),\theta_{4}\left(\tau \right)$ are elliptic theta functions. $K_{2p}^{0,\frac{1}{2}}\left(\tau \right)$ is Kronecker’s double series [26,50].

The differential operators $\Lambda_{2p}$ that appear here can be expressed via coefficients $z_{2p}$ of the Taylor expansion of the lattice dispersion relation $\omega_{z}\left(x\right)$

(A6) $$\omega_{z}\left(x\right)=x\left(\sqrt{z}+\sum_{p=1}^{\infty }\frac{z_{2p}}{(2p)!}x^{2p}\right),$$

with $z_{2}=-\sqrt{z}\left(1+z\right)/3$, $z_{4}=\sqrt{z}\left(1+z\right)\left(1+9z\right)/5$, $z_{6}=-\sqrt{z}\left(1+z\right)\left(1+90z+225z^{2}\right)/7$, etc.

$$\begin{array}{cccc} \Lambda_{2} & = & z_{2}, & \\ \Lambda_{4} & = & z_{4}+3z_{2}^{2}\;\frac{\partial }{\partial z}, & \\ \Lambda_{6} & = & z_{6}+15z_{4}z_{2}\;\frac{\partial }{\partial z}+15z_{2}^{3}\;\frac{\partial^{2}}{\partial z^{2}}, & \left(\text{A7}\right)\\ & \vdots & & \\ \Lambda_{p} & = & \sum_{r=1}^{p}\sum {\left(\frac{z_{p_{1}}}{p_{1}!}\right)}^{k_{1}}\ldots {\left(\frac{z_{p_{r}}}{p_{r}!}\right)}^{k_{r}}\frac{p!}{k_{1}!\ldots k_{r}!}\;\frac{\partial^{k}}{\partial z^{k}}. & \;\;\;\;\;\;\;\;(\text{A8}) \end{array}$$

Here, summation is over all positive numbers $\left\{k_{1}\ldots k_{r}\right\}$ and different positive numbers $\left\{p_{1},\ldots ,p_{r}\right\}$ such that $p_{1}k_{1}+\ldots +p_{r}k_{r}=p$ and $k=k_{1}+\ldots +k_{r}-1$.

The $\int_{0}^{\pi }\;\;\omega_{z}\left(x\right)\;\;dx$ is given by

(A9) $$\int_{0}^{\pi }\;\;\omega_{z}\left(x\right)\;\;dx=\frac{z^{1/2}\;\Phi \left(-z,2,\frac{1}{2}\right)}{2},$$

where Φ(x,s,α) is the Lerch transcendent defined as

(A10) $$\Phi \left(x,s,\alpha \right)=\sum_{n=0}^{\infty }{\left(\alpha +n\right)}^{-s}x^{n}.$$

In particular, for the isotropic spanning tree (z=1), the Lerch transcendent is now Φ(−1,2,1/2)=4G, where *G* is the Catalan constant given by

(A11) $$G=\sum_{n=0}^{\infty }\frac{{\left(-1\right)}^{n}}{{\left(2n+1\right)}^{2}}=0.915965594\ldots .$$

Kronecker’s double series $K_{2p}^{0,\frac{1}{2}}\left(\tau \right)$ can all be expressed in terms of the elliptic θ-functions only [26].
